# Genome Sequences of Two Novel Papillomaviruses Isolated from Healthy Skin of *Pudu puda* and *Cervus elaphus* Deer

**DOI:** 10.1128/genomeA.00298-18

**Published:** 2018-05-03

**Authors:** Beatriz Mengual-Chuliá, Ulrich Wittstatt, Philipp Olias, Ignacio G. Bravo

**Affiliations:** a Infections and Cancer Laboratory, Catalan Institute of Oncology (ICO), Barcelona, Spain; b Department of Animal Diseases, Zoonoses and Infection Diagnostic, Berlin-Brandenburg State Laboratory, Berlin, Germany; c Institute of Animal Pathology, University of Bern, Bern, Switzerland; d Centre National de la Recherche Scientifique (CNRS), Laboratory MIVEGEC (UMR CNRS IRD UM), Montpellier, France

## Abstract

We report the complete genome sequences of Pudu puda papillomavirus *1* (PpudPV1) and Cervus elaphus papillomavirus *2* (CelaPV2), isolated from healthy skin hair follicles of a Southern pudu and a red deer, respectively. PpudPV1 is basal to the DyokappaPVs, whereas CelaPV2 is basal to the XiPVs (Beta-XiPV crown group).

## GENOME ANNOUNCEMENT

Papillomaviruses (PVs) are small nonencapsidated viruses with a circular double-stranded DNA genome of ca. 8 kbp in length. PVs infect epithelia in most amniotes, causing asymptomatic infections, proliferative benign lesions, and different cancers (1, 2).

Two adult deer living in captivity in zoos in Berlin, Germany, were sampled for the search of novel animal PVs. One of the specimens was a female Southern pudu (Pudu puda; Mammalia: Artiodactyla: Cervidae: Odocoileinae: Pudu). The Southern pudu is native to Argentina and Chile and is classified as “vulnerable” on the International Union for Conservation of Nature (IUCN) Red List of Threatened Species (http://www.iucnredlist.org/). The second specimen was a female red deer (Cervus elaphus Mammalia: Artiodactyla: Cervidae: Odocoileinae: Cervus). The red deer has a large global distribution extending from Europe and North Africa through central Asia, Siberia, the Far East and North America, and the IUCN Red List classifies this species as of “least concern.”

Hair follicles from the healthy skin of each animal were collected and tested for the presence of PV DNA using a battery of broad-spectrum PCR primers against the *E1* and *L1* genes (3). This partial information (ca. 450 bp) was used to design tail-to-tail primers to amplify the full-length genome using long-range PCR. The amplicons were Sanger sequenced in both strands through primer walking and cloned. Phylogenetic relationships for the *E1E2*, *L2L1*, and *E1E2L2L1* gene concatenates were inferred under a maximum likelihood framework (4).

Both P. puda papillomavirus *1* (PpudPV1) and C. elaphus papillomavirus *2* (CelaPV2) genomes show the classical PV genome arrangement: the upstream regulatory region (URR), the early genes *E6*, *E7*, *E1*, and *E2*, and the late genes *L2* and *L1*. The PpudPV1 *L1* gene shares similar levels of nucleotide identity with very divergent PVs: 66% with the giant panda Ailuropoda melanoleuca papillomavirus *1* (AmPV1; Alpha-OmicronPV crown group: Omega PVs), 66% with the bottlenose dolphin Tursiops truncatus papillomavirus *8* (TtPV8; Alpha-OmicronPV crown group, DyopiPVs), and 65% with the human PV 204 (HPV204; Kappa-LambdaPV crown group, MuPV). In accordance with the International Committee on Taxonomy of Viruses (ICTV) recommendations, this viral genome is thus of *incertae sedis*, clearly showing the need for strong revision of the current taxonomic criteria (5, 6). However, phylogeny confidently places PpudPV1 basal to the clade consisting of BPV18 and the DyokappaPVs. All members of this clade have been isolated from Caprinae (Rupicapra rupicapra [7] and Ovis aries [8]) and Bovinae species (Bos taurus [9, 10]), mostly from benign neoplasias. This PV clade does not belong to any of the four large PV crown groups (11).

The CelaPV2 *L1* gene shares between 67 and 69% nucleotide identity with different PVs in the XiPVs genus, all of them isolated from Bovidae and Cervidae species, which is in good agreement with the phylogenetic placement. CelaPV2 belongs thus in the Beta-XiPV crown group and is only distantly related to C. elaphus papillomavirus *1* (Delta-ZetaPV crown group, EpsilonPVs), isolated from a red deer fibropapilloma (12, 13).

This study expands the known diversity of PVs infecting cervids and highlights the multiple evolutionary origins of PVs infecting cetartiodactyls.

### Accession number(s).

The complete genome sequences for PpudPV1 and for CelaPV2 are available in GenBank under the accession numbers KT932713 and KT932712, respectively.

## References

[1] Van Doorslaer K 2013 Evolution of the *Papillomaviridae*. Virology 445:11–20. doi:10.1016/j.virol.2013.05.012. 23769415

[2] Bravo IG, Félez-Sánchez M 2015 Papillomaviruses: viral evolution, cancer and evolutionary medicine. Evol Med Public Health 2015:32–51. doi:10.1093/emph/eov003. 25634317 PMC4356112

[3] Mengual-Chuliá B, García-Pérez R, Gottschling M, Nindl I, Bravo IG 2012 Novel animal papillomavirus sequences and accurate phylogenetic placement. Mol Phylogenet Evol 65:883–891. doi:10.1016/j.ympev.2012.08.011. 22960206

[4] Stamatakis A 2014 RAxML version 8: a tool for phylogenetic analysis and post-analysis of large phylogenies. Bioinformatics 30:1312–1313. doi:10.1093/bioinformatics/btu033. 24451623 PMC3998144

[5] Bravo IG, de Sanjosé S, Gottschling M 2010 The clinical importance of understanding the evolution of papillomaviruses. Trends Microbiol 18:432–438. doi:10.1016/j.tim.2010.07.008. 20739182

[6] Bravo IG, de Sanjosé S, Gottschling M 2011 Papillomaviruses and Darwinian classification: response to Van Doorslaer et al. Trends Microbiol 19:49–50. doi:10.1016/j.tim.2010.11.008.21144754

[7] Mengual-Chuliá B, Domenis L, Robetto S, Bravo IG 2014 A novel papillomavirus isolated from a nasal neoplasia in an Italian free-ranging chamois (*Rupicapra r. rupicapra*). Vet Microbiol 172:108–119. doi:10.1016/j.vetmic.2014.05.006. 24910075

[8] Alberti A, Pirino S, Pintore F, Addis MF, Chessa B, Cacciotto C, Cubeddu T, Anfossi A, Benenati G, Coradduzza E, Lecis R, Antuofermo E, Carcangiu L, Pittau M 2010 *Ovis aries* papillomavirus 3: a prototype of a novel genus in the family *Papillomaviridae* associated with ovine squamous cell carcinoma. Virology 407:352–359. doi:10.1016/j.virol.2010.08.034. 20863546

[9] Daudt C, da Silva FRC, Streck AF, Weber MN, Mayer FQ, Cibulski SP, Canal CW 2016 How many papillomavirus species can go undetected in papilloma lesions? Sci Rep 6:36480. doi:10.1038/srep36480. 27808255 PMC5093584

[10] Bauermann FV, Joshi LR, Mohr KA, Kutish GF, Meier P, Chase C, Christopher-Hennings J, Diel DG 2017 A novel bovine papillomavirus type in the genus *Dyokappapapillomavirus*. Arch Virol 162:3225–3228. doi:10.1007/s00705-017-3443-9. 28616671

[11] Gottschling M, Göker M, Stamatakis A, Bininda-Emonds ORP, Nindl I, Bravo IG 2011 Quantifying the phylodynamic forces driving papillomavirus evolution. Mol Biol Evol 28:2101–2113. doi:10.1093/molbev/msr030. 21285031

[12] Scagliarini A, Gallina L, Battilani M, Turrini F, Savini F, Lavazza A, Chiari M, Coradduzza E, Peli A, Erdélyi K, Alberti A 2013 *Cervus elaphus* papillomavirus (CePV1): new insights on viral evolution in deer. Vet Microbiol 165:252–259. doi:10.1016/j.vetmic.2013.03.012. 23578708

[13] Erdélyi K, Gál J, Sugár L, Ursu K, Forgách P, Szeredi L, Steineck T 2009 Papillomavirus-associated fibropapillomas of red deer (*Cervus elaphus)*. Acta Vet Hung 57:337–344. doi:10.1556/AVet.57.2009.2.14. 19584046

